# Prenatal maternal antidepressants, anxiety, and depression and offspring DNA methylation: epigenome-wide associations at birth and persistence into early childhood

**DOI:** 10.1186/s13148-019-0653-x

**Published:** 2019-03-29

**Authors:** Andres Cardenas, Sabrina Faleschini, Andrea Cortes Hidalgo, Sheryl L. Rifas-Shiman, Andrea A. Baccarelli, Dawn L. DeMeo, Augusto A. Litonjua, Alexander Neumann, Janine F. Felix, Vincent W. V. Jaddoe, Hanan El Marroun, Henning Tiemeier, Emily Oken, Marie-France Hivert, Heather H. Burris

**Affiliations:** 1 Division of Environmental Health Sciences, School of Public Health, University of California, Berkeley, Berkeley, CA USA; 2 School of Psychology, Laval University, Quebec, QC Canada; 3 Department of Child and Adolescent Psychiatry and Psychology, Erasmus MC – Sophia Children’s Hospital, Rotterdam, the Netherlands; 4 Division of Chronic Disease Research Across the Lifecourse, Department of Population Medicine, Harvard Medical School and Harvard Pilgrim Health Care Institute, Boston, MA USA; 5 Department of Environmental Health Sciences, Mailman School of Public Health, Columbia University, New York, NY USA; 6 Channing Division of Network Medicine, Department of Medicine, Brigham and Women’s Hospital, Harvard Medical School, Boston, MA USA; 7 Division of Pediatric Pulmonary Medicine, University of Rochester Medical Center, Rochester, NY USA; 8 Generation R Study Group, Erasmus MC, University Medical Center Rotterdam, Rotterdam, the Netherlands; 9 Department of Epidemiology, Erasmus MC, University Medical Center Rotterdam, Rotterdam, the Netherlands; 10 Department of Pediatrics, Erasmus MC, University Medical Center Rotterdam, Rotterdam, the Netherlands; 11 Diabetes Unit, Massachusetts General Hospital, Boston, MA USA; 12 Division of Neonatology, Department of Pediatrics, Children’s Hospital of Philadelphia and Perelman School of Medicine, University of Pennsylvania, Philadelphia, PA USA

**Keywords:** Maternal depression, Maternal anxiety, Antidepressants, DNA methylation, Fetal programming

## Abstract

**Background:**

Maternal mood disorders and their treatment during pregnancy may have effects on the offspring epigenome. We aim to evaluate associations of maternal prenatal antidepressant use, anxiety, and depression with cord blood DNA methylation across the genome at birth and test for persistence of associations in early and mid-childhood blood DNA.

**Methods:**

A discovery phase was conducted in *Project Viva*, a prospective pre-birth cohort study with external replication in an independent cohort, the *Generation R Study*. In Project Viva, pregnant women were recruited between 1999 and 2002 in Eastern Massachusetts, USA. In the Generation R Study, pregnant women were recruited between 2002 and 2006 in Rotterdam, the Netherlands. In Project Viva, 479 infants had data on maternal antidepressant use, anxiety, depression, and cord blood DNA methylation, 120 children had DNA methylation measured in early childhood (~ 3 years), and 460 in mid-childhood (~ 7 years). In the Generation R Study, 999 infants had data on maternal antidepressants and cord blood DNA methylation. The prenatal antidepressant prescription was obtained from medical records. At-mid pregnancy, symptoms of anxiety and depression were assessed with the Pregnancy-Related Anxiety Scale and the Edinburgh Postnatal Depression Scale in Project Viva and with the Brief Symptom Inventory in the Generation R Study. Genome-wide DNA methylation was measured using the Infinium HumanMethylation450 BeadChip in both cohorts.

**Results:**

In Project Viva, 2.9% (14/479) pregnant women were prescribed antidepressants, 9.0% (40/445) experienced high pregnancy-related anxiety, and 8.2% (33/402) reported symptoms consistent with depression. Newborns exposed to antidepressants in pregnancy had 7.2% lower DNA methylation (95% CI, − 10.4, − 4.1; *P* = 1.03 × 10^−8^) at cg22159528 located in the gene body of *ZNF575*, and this association replicated in the Generation R Study (*β* = − 2.5%; 95% CI − 4.2, − 0.7; *P* = 0.006). In Project Viva, the association persisted in early (*β* = − 6.2%; 95% CI − 10.7, − 1.6) but not mid-childhood. We observed cohort-specific associations for maternal anxiety and depression in Project Viva that did not replicate.

**Conclusions:**

The *ZNF575* gene is involved in transcriptional regulation but specific functions are largely unknown. Given the widespread use of antidepressants in pregnancy, as well as the effects of exposure to anxiety and depression, implications of potential fetal epigenetic programming by these risk factors and their impacts on development merit further investigation.

**Electronic supplementary material:**

The online version of this article (10.1186/s13148-019-0653-x) contains supplementary material, which is available to authorized users.

## Background

Anxiety and depression are common during pregnancy, affecting up to 8% and 12% of pregnant women, respectively [1–3]. Prenatal anxiety and depression are associated with poor perinatal outcomes including suboptimal fetal growth [4, 5] and preterm birth [6]. While generally thought to be safe, medications to treat mood disorders in pregnancy have been associated with risks of adverse long-term consequences for children including impaired neuromotor development [7] as well as behavioral and emotional problems [8–11].

Antidepressants such as selective serotonin reuptake inhibitors (SSRIs) are used to reduce symptoms of anxiety and depression in approximately 8% of US pregnant women [12]. Two recent reviews of the literature found that fetuses exposed to antidepressants such as SSRIs may have abnormal motor and heart rate activity during fetal development [13, 14]. It is well established that early-life environments may influence fetal and later child development [15]. Epigenetic processes during fetal development are one pathway by which environmental factors may affect phenotype later in life [16]. Whether antidepressants or the underlying psychopathology in pregnancy affects fetal programming through epigenetic processes such as DNA methylation remains unknown.

Epigenome-wide association studies (EWAS) can be a powerful tool to discover biomarkers of disease and to understand biologic processes [17]. Using an epigenome-wide approach, we aimed to identify differences in DNA methylation in neonates associated with prenatal maternal antidepressant use, anxiety, and depression. We hypothesized that prenatal maternal exposure to antidepressants, anxiety, and depression would lead to differences in DNA methylation in cord blood that would persist into childhood.

## Results

Overall, in Project Viva, 2.9% (14/479) of women were prescribed antidepressants during pregnancy, 9.0% (40/445) experienced high pregnancy-related anxiety, and 8.2% (33/402) reported symptoms consistent with depression in pregnancy. In the Generation R Study, there were 999 mother-infant pairs eligible for analyses, 1.4% (14/999) were prescribed antidepressants, 5.8% (56/969) experienced high anxiety, and 3.2% (31/969) reported symptoms consistent with clinical depression in pregnancy. Demographic characteristics of participants in both cohorts are presented in Table 1.

**Table 1:** Characteristics of the discovery cohort, Project Viva, and the independent replication cohort, Generation R Study

Characteristics	Discovery cohort Project Viva *N* = 479		Replication cohort Generation R Study *N* = 999	
Maternal	Mean (SD) or *n* (%)			
Age, years	32.1 (5.4)		32.2 (4.3)	
Pre-pregnancy BMI, kg/m^2^	24.7 (5.2)		23.4 (3.9)	
Antidepressant use	14 (2.9%)		14 (1.4%)	
High anxiety	40 (9.0%)^a^		56 (5.8%)^b^	
Depression mid-pregnancy	33 (8.2%)^a^		31 (3.2%)^b^	
Race/ethnicity				
	Non-Hispanic White	341 (71.2%)	Dutch	931 (93.2%)
	Non-Hispanic Black	56 (11.7%)	Non-Dutch Western	63 (6.3%)
	Hispanic	37 (7.7%)	Non-western	5 (0.5%)
	Other	45 (9.4%)	–	
College graduate or more education	317 (66.2%)		669 (67.0%)	
Smoking status				
	Never	327 (68.3%)	Never during pregnancy	772 (77.3%)
	Former	100 (20.9%)	Quit when pregnancy was known	93 (9.3%)
	During pregnancy	52 (10.9%)	Continued during pregnancy	134 (13.4%)
Perinatal/infant	Mean (SD) or *n* (%)			
Cesarean delivery	79 (16.5%)		103 (10.3%)	
Gestational age at delivery, weeks	39.8 (1.4)		40.2 (1.4)	
Birth weight-for-gestational age, *z-*score	0.27 (1.0)		0.26 (0.87)^c^	
Female infant	229 (47.8%)		484 (48.4%)	

^a^34 missing data on maternal anxiety and 77 missing data on maternal depression in Project Viva

^b^86 missing data on maternal depression and anxiety in the Generation R Study

^c^1 missing data for birthweight-for-gestational age in Generation R Study

**Fig. 1:**
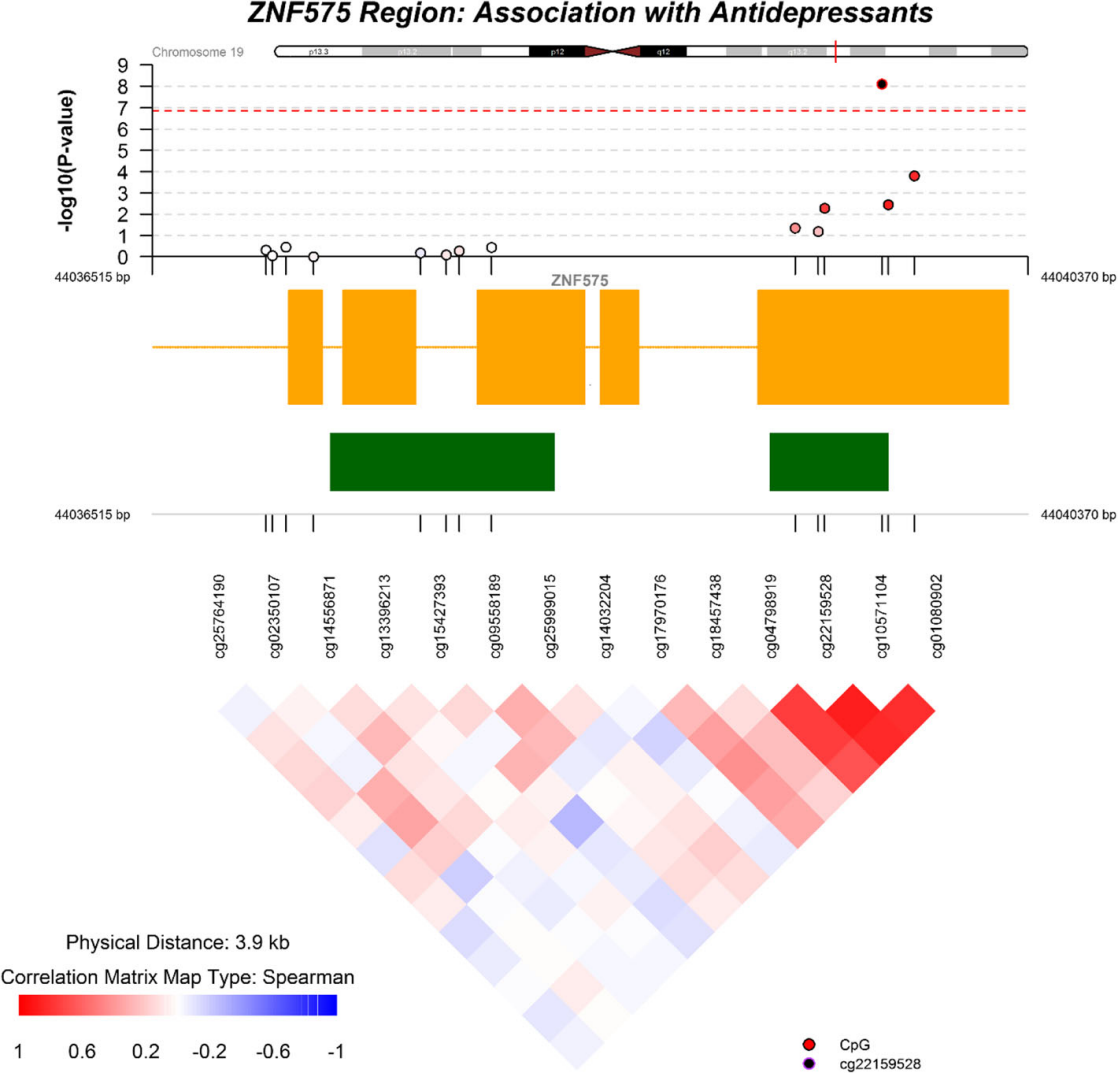
Regional Manhattan plot for the adjusted association of prenatal maternal antidepressants and umbilical cord blood DNA methylation within *ZNF575* gene region in Project Viva (orange squares indicate exons; orange lines indicate introns; green squares indicate CpG islands)

In Project Viva, exposure to antidepressants was associated with DNA methylation differences at 130 CpG sites that passed FDR < 0.05, among which 16 sites also passed Bonferroni significance (*P* < 1.34 × 10^−7^) in models adjusted for maternal, parity, self-reported race, smoking during pregnancy, body mass index (BMI), mode of delivery, education and infant sex, gestational age at birth, and nucleated cell-type proportions (Table 2). In replication analyses in the Generation R Study, among Bonferroni significant sites discovered in Project Viva, we confirmed that DNA methylation of one of these sites, cg22159528, was significantly lower among infants whose mothers were prescribed antidepressants during pregnancy. This CpG site is located in the body of the Zinc Finger Protein 575 gene (*ZNF575*) on chromosome 19 and annotated to a CpG island. Specifically, in Project Viva, we observed that infants born to mothers prescribed antidepressant in pregnancy had 7.2% lower DNA methylation (95% CI − 10.4, − 4.1; *P* = 1 × 10^−8^) at this site and in the Generation R Study, exposed infants had 2.5% lower DNA methylation (95% CI − 4.2, − 0.7; *P* = 0.006) at the same site in adjusted models. In the discovery cohort, we also observed an additional four CpG sites (cg01080902, cg04798919, cg10571104, and cg17970176) near cg22159528 in the *ZNF575* gene that were nominally associated with antidepressant use (*P* < 0.05) in the replication cohort but did not reach a Bonferroni adjusted (0.05/16) levels of significance (Fig. 1). One other CpG site in the replication cohort (cg00367463; *BEST4* gene) passed the *P* < 0.05 criteria for replication but its effect estimate was in the opposite direction.

**Table 2:** Differentially methylated CpGs in umbilical cord blood DNA associated with prenatal maternal antidepressants in pregnancy

CpG	Chr	Genomic Position	Gene	Discovery cohort Project Viva (*n* = 479)			Replication cohort Generation R Study (*n* = 999^c^)		
				Mean (SD) %-DNA methylation	Adjusted % change in DNA methylation (95% CI)^a^	*P*	Mean (SD) %-DNA methylation	Adjusted % change in DNA methylation (95% CI)^b^	*P*
cg00367463	1	45,249,899	*BEST4*	1.9 (0.4)	0.26 (0.16, 0.37)	2.01 × 10^−8^	10.3 (2.7)	− 0.53 (− 1.02, − 0.03)	0.04
cg27566858	1	208,084,099	*CD34*	2.0 (0.3)	0.27 (0.15, 0.39)	3.13 × 10^−9^	12.6 (3.3)	0.26 (− 0.53, 1.06)	0.52
cg03536711	1	221,509,067	*LOC400804*	53.3 (11.4)	− 11.63 (− 15.80, −7.45)	3.24 × 10^−8^	54.9 (9.3)	2.25 (− 1.53, 6.02)	0.24
cg07729367	3	128,479,008	*RAB7A*	98.2 (0.5)	− 0.49 (− 0.70, − 0.27)	1.08 × 10^−7^	90.6 (2.1)	− 0.31 (− 1.36, 0.75)	0.57
cg22065513	3	144,241,532		97.1 (1.4)	− 1.22 (− 1.73, − 0.72)	4.11 × 10^−9^	90.7 (2.0)	− 0.36 (− 1.33, 0.61)	0.47
cg27299660	3	171,527,797	*PLD1*	1.7 (0.4)	0.77 (0.49, 1.04)	1.19 × 10^−7^	8.3 (2.3)	0.11 (− 0.55, 0.77)	0.75
cg14499053	7	19,158,954		2.2 (0.5)	− 0.33 (− 0.45, − 0.22)	5.44 × 10^−8^	11.5 (2.1)	0.35 (− 0.48, 1.19)	0.41
cg15881597	7	73,085,754	*VPS37D*	40.1 (4.9)	− 3.64 (− 5.09, − 2.19)	5.66 × 10^−9^	47.3 (4.7)	− 1.82 (− 4.06, 0.42)	0.11
cg06645921	12	8,025,394	*SLC2A14*	6.6 (3.7)	− 2.38 (− 2.95, − 1.81)	9.11 × 10^−16^	*NA*	*NA*	*NA*
cg27161197	12	47,224,649		71.5 (5.9)	− 5.17 (− 7.12, − 3.23)	1.77 × 10^−9^	68.9 (5.5)	− 2.01 (− 5.23, 1.21)	0.22
cg25121621	15	45,926,780	*SQRDL*	19.6 (2.9)	2.74 (1.82, 3.67)	2.71 × 10^−12^	28.4 (4.2)	− 0.80 (− 4.37, 2.78)	0.66
cg06358612	17	28,619,293	*BLMH*	1.4 (0.3)	0.22 (0.14, 0.31)	6.07 × 10^−8^	8.1 (1.4)	− 0.18 (− 0.71, 0.34)	0.49
*cg22159528*	*19*	*44,039,727*	*ZNF575*	*51.0 (6.8)*	*− 7.23 (− 10.36, − 4.10)*	*1.03 × 10* ^*−8*^	*54.7 (5.9)*	*− 2.46 (− 4.23, − 0.69)*	*0.006*
cg12489353	19	48,231,499	*EHD2*	79.3 (14.5)	− 10 (− 15.7, − 4.3)	7.88 × 10^−8^	77.2 (10)	− 0.80 (− 6.49, 4.88)	0.78
cg11449935	20	35,202,477	*TGIF2*	2.2 (0.4)	0.29 (0.16, 0.43)	4.20 × 10^−8^	10.2 (2.2)	0.31(− 0.52, 1.13)	0.47
cg18036763	22	45,404,910	*PHF21B*	10.2 (3.6)	3.24 (1.75, 4.74)	7.62 × 10^−8^	6.2 (1.7)	0.38 (− 0.34, 1.11)	0.30

*Abbreviations*: *SD* standard deviation, *CI* confidence interval, *NA* excluded from the replication cohort (Generation R Study) after standard quality control

^a^Adjusted for maternal age, parity, race/ethnicity, smoking (never, former and during pregnancy), pre-pregnancy BMI, mode of delivery, education and infant sex, gestational age, and estimated cord blood nucleated cells (CD8, CD4, Mono, NK, B cells, granulocytes and nRBCs)

^b^Adjusted for the same covariates as above and included sample plate as an additional covariate

^c^Refers to sample size for cg22159528, whereas other models from Generation R Study excluded samples that failed at specific CpG sites (smallest sample size *n* = 970 for cg18036763)

In Project Viva, we observed 13 individual CpG sites differentially methylated relative to high maternal pregnancy-related anxiety and three individual sites associated with prenatal maternal depression (FDR < 0.05) but these associations were not robust to external replication in the Generation R Study (Additional file 1: Table S1). For single CpG analyses, the genomic inflation factor (*λ*) was 0.87 for prenatal antidepressants, 1.17 for high anxiety, and 0.94 for depression indicating a reasonable fit (Additional file 2: Figure S1). As a secondary approach, we conducted regional analyses using DMRcate: we did not find any differentially methylated regions relative to prenatal antidepressant prescription, anxiety, or depression in the discovery cohort.

**Fig. 2:**
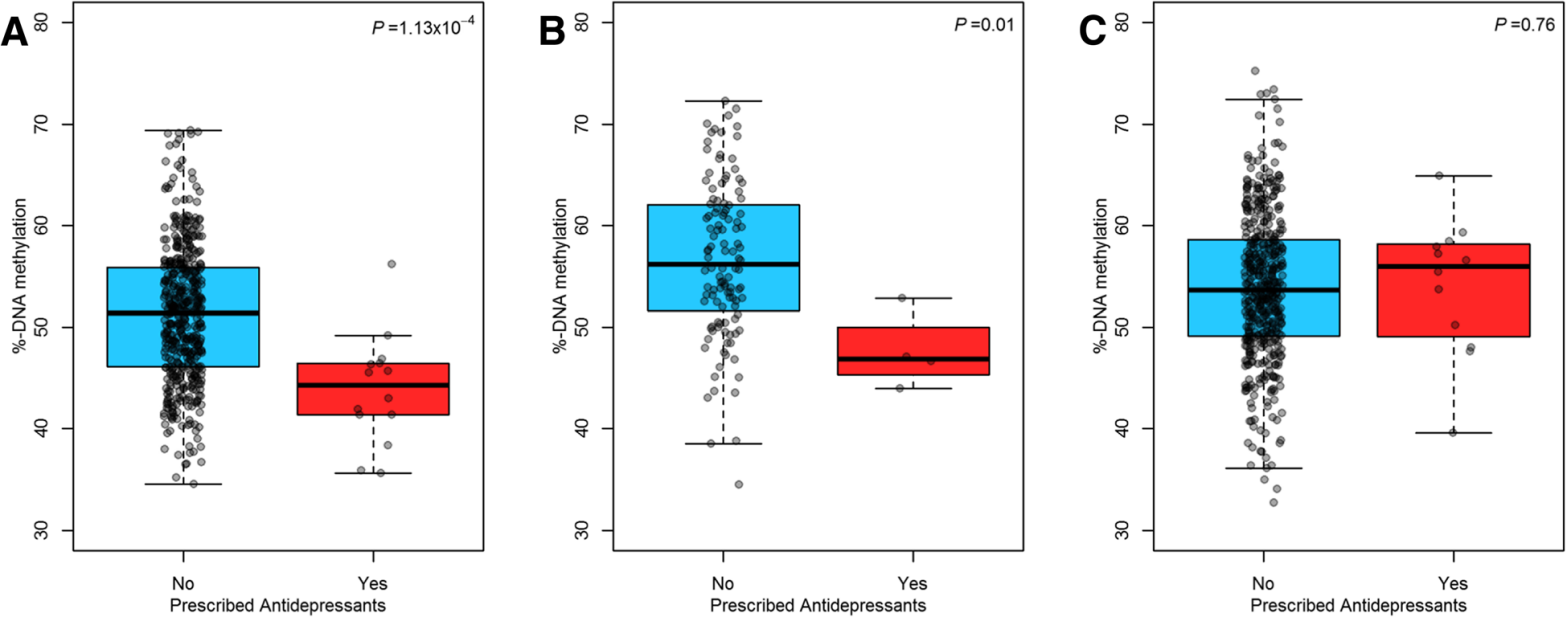
Unadjusted %-DNA methylation distribution for antidepressants exposed and unexposed infants at the replicated CpG site (cg22159528) in the *ZNF575* gene and unadjusted Wilcoxon-rank sum test *P* value in the discovery cohort, Project Viva, measured at three time points: **a** umbilical cord blood (*n* = 479), **b** early childhood (*n* = 120), and **c** mid-childhood peripheral blood (*n* = 460). One hundred twelve participants in early childhood also had cord blood measurements, and 235 participants from mid-childhood also had cord blood measurements

We evaluated the persistence of the observed association at cg22159528 in the *ZNF575* gene for antidepressants and DNA methylation in Project Viva, in blood collected in early and mid-childhood. In adjusted models, prenatally exposed children (*n* = 4 out of 120) had 6.2% lower DNA methylation (95% CI − 10.7 to − 1.6; *P* = 6.70 × 10^−3^) compared to non-exposed children in early childhood. This association was in the same direction but attenuated and non-significant in mid-childhood (*β* = − 3.7, 95% CI − 8.8 to 1.4; *P* = 0.16) (*n* = 12 exposed out of 460). Unadjusted differences in DNA methylation were similar to adjusted differences for exposed and unexposed infants at birth, early, and mid-childhood (Fig. 2).

**Fig. 3:**
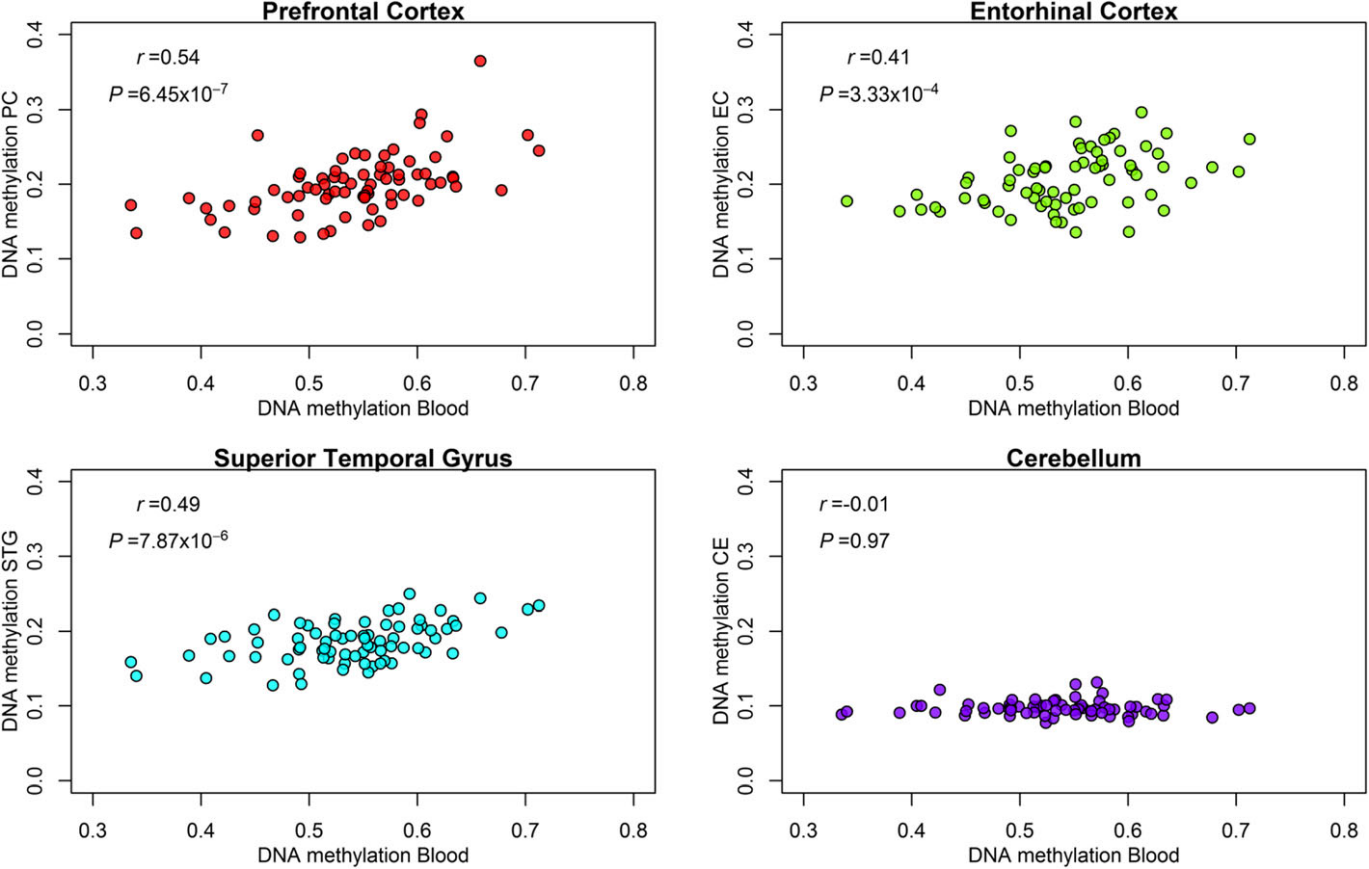
Scatterplots and correlations for cg22159528 (*ZNF575* gene) methylation levels of blood DNA and four brain regions: prefrontal cortex (PC, *n* = 74), entorhinal cortex (EC, *n* = 71), superior temporal gyrus (STG, *n* = 75), and cerebellum (CE, *n* = 71). Samples from external paired dataset of blood and brain tissue of adults [51].

To evaluate the potential neurological implications of our findings, we tested correlations between blood and brain DNA methylation using external reference data. DNA methylation at cg22159528 in the *ZNF575* gene from over 70 adults showed positive correlations between blood and brain tissue of the prefrontal cortex (*r* = 0.54, *P* = 6.45 × 10^−7^), entorhinal cortex (*r* = 0.41, *P* = 2.33 × 10^−4^), superior temporal gyrus (*r* = 0.49, *P* = 7.87 × 10^−8^) but not the cerebellum (*r* = − 0.01, *P* = 0.97) (Fig. 3). These results must be interpreted with caution given that reference blood and brain samples were collected from adults and might not accurately reflect variation in cord blood or early childhood blood samples with brain DNA methylation.

## Discussion

Using an agnostic epigenome-wide approach, we observed differences in DNA methylation across multiple CpG sites for infants prenatally exposed to maternal antidepressants and replicated this observation at one CpG site. While 13 CpG sites were associated with high maternal prenatal anxiety and three with prenatal maternal depression in Project Viva, we did not confirm these associations in the Generation R Study. In both Project Viva and Generation R Study, antidepressant prescription during pregnancy was associated with lower DNA methylation at a CpG site located within the *ZNF575* gene body. Exposure to antidepressants during pregnancy was also associated with lower DNA methylation at this site in early childhood blood with a similar magnitude of the effect. Using a blood and brain DNA methylation reference database, we observed moderate correlations among three brain regions with blood cells at the discovered and validated CpG site in the *ZNF575* gene.

The Zinc Finger Protein 575 gene (*ZNF575*) is part of a large family of zinc finger proteins with multiple diverse functions that are abundant across multiple eukaryotic genomes [18]. This protein is involved in transcriptional regulation and has been previously associated with lung cancer [19]. Otherwise, there is very little known about the function of the *ZNF575* gene and its role in health or development. This top finding was persistent in early, at approximately 3 years of age, but not in mid-childhood in the discovery cohort. This is important, as the first 1000 days of life represent a period of rapid development and vulnerability able to influence the life course further stressing the need to fully characterize the function of the *ZNF575* gene*.*

There were an additional 15 CpG sites in cord blood associated with maternal antidepressant use that survived Bonferroni correction in Project Viva, but they were not replicated in The Generation R Study. Six prior studies of in utero antidepressant exposure and offspring DNA methylation were recently systematically reviewed by Viuff et al. [20]. The authors concluded that there was no consistent association among studies and highlighted the need for untargeted epigenetic assays with external validation [20]. None of the prior studies reported differentially methylated sites at/near *ZNF575*. Three of the studies used a candidate gene approach [21–23]; two used an earlier epigenome-wide array which analyzed only 27,000 CpG sites [24, 25]. Only one prior study by Non et al. examined associations between maternal SSRI use and offspring DNA methylation using the same DNA methylation platform as we did [26]. They used a case-control design of 22 exposed infants and 23 unexposed infants and found no significant association between SSRIs and offspring DNA methylation. In addition, Non and colleagues selected infants exposed to SSRIs that differ from the medications used in our population and did not adjust for cell-type composition. Lack of consistency found in the literature may be a result of differences in study design, population, technology for DNA methylation assessment, and smaller sample sizes as well as exposure timing and ascertainment.

A few of the cohort-specific associations of high levels of pregnancy-related anxiety with DNA methylation in Project Viva were consistent with prior literature. For example, a CpG site in the glial cell-derived neurotrophic factor (*GDNF*) gene showed higher DNA methylation compared to low or moderate pregnancy-related anxiety. Using blood samples, a study on inflammatory markers of women with antenatal depression found DNA methylation at another CpG site near *GDNF* to be higher among depressed pregnant women [27]. Also, in a mouse model, DNA methylation of the *GDNF* gene in experimentally stressed mice has been shown to be differentially methylated relative to stress. These experiments also showed that chronic stress reduced levels of a histone modification, H3K4me3, in the promoter region of the *GDNF* gene and this effect was reversed by antidepressants [28].

Our study has several limitations. In Project Viva, over seven types of antidepressants were used during pregnancy with some women prescribed more than one single type, although 12/14 were SSRIs (Additional file 1: Table S2). In the Generation R Study, antidepressant prescriptions were limited to tricyclic antidepressants and SSRIs. In addition, in the Generation R Study, general anxiety during pregnancy was ascertained while pregnancy-related anxiety was evaluated in Project Viva. These are different scales and could capture different sources and levels of anxiety. Moreover, we measured DNA methylation only in blood and it is likely that blood may not accurately reflect DNA methylation variability in other relevant tissues. However, we used external brain and blood reference DNA methylation data to compare correlations at the externally replicated site. Our hypothesis was based on DNA methylation programming during fetal development. However, another possibility is that of cellular polycreodism—or the systematic variability of cell fate to yield a distinctive repertoire of cells [29]. Yet, without experimental data, it is impossible to determine the true causal effects of these exposures in the epigenome and therefore results should be interpreted as biomarkers. It would be nearly impossible to conduct a randomized trial for these prenatal maternal exposures and conditions. Additionally, timing, severity, and accuracy of self-reported depression and pregnancy-related anxiety along with medication adherence for antidepressants and repeated exposure could introduce substantial exposure misclassification making it challenging to capture underlying associations. Further, any study of the effects of medications can be affected by confounding by indication. Specifically, it may be the most depressed or anxious women who were treated with antidepressants and that those underlying causes were truly responsible for the observed associations. This issue is further complicated by the small overlap of women who exhibited symptoms of anxiety (*n* = 2) or depression (*n* = 4) among the treated women in Project Viva. The two cohorts also differed from one another, especially with respect to ethnicity (Table 1), which may have limited our ability to replicate findings across different populations. Lastly, our antidepressant-exposed sample was small, limiting statistical power.

Our study also has important strengths. First, we implemented an epigenome-wide approach to agnostically capture associations with a relatively large sample size. Our prospective design reduces the chance of bias that might arise from case-control studies and allowed us to collect valuable confounder information early during pregnancy. Another major strength is the replication of findings in an independent birth cohort. Our use of an external reference dataset demonstrated moderate to strong correlations between DNA methylation of blood and three brain regions in the replicated site (*ZNF575*), suggesting that this finding may be relevant to long-term mental health or neurodevelopment. Yet, more work is needed to fully characterize the function of the *ZNF575* gene*.*

## Conclusion

In conclusion, we found DNA methylation of the *ZNF575* gene in infant cord blood to be associated with maternal antidepressant use in pregnancy in two independent cohorts. We also demonstrated that this association persists into early childhood. These findings warrant further study to confirm the association and to determine its clinical significance.

## Methods

### Discovery cohort: Project Viva

We studied mother-child pairs participating in Project Viva, a prospective pre-birth cohort study recruited between 1999 and 2002 from Atrius Harvard Vanguard Medical Associates in MA, USA [30]. Mothers provided written, informed consent, and the institutional review board of Harvard Pilgrim Health Care approved the study. Of the total 2128 singleton births, there were 485 infants with cord blood DNA methylation data and information on prenatal maternal antidepressants, anxiety, and depression. We excluded 6 infants with gestational age < 34 weeks and analyzed 479 mother-infant pairs with cord blood DNA methylation. We evaluated persistence of epigenetic associations observed at birth in 120 children (*n* = 112 included in cord blood analyses) with peripheral blood DNA methylation measurements from early childhood (mean 3.4 years, range 2.9 to 5.3) and 460 children (*n* = 235 included in cord blood analyses) with peripheral blood DNA samples from mid-childhood (mean 7.9 years, range 6.7 to 10.5).

We defined women as exposed to antidepressants if the medical record included a prescription during pregnancy (Additional file 1: Table S2). To assess anxiety, at the mid-pregnancy visit, we administered the 7-item Pregnancy-Related Anxiety Scale (PRAS) [31]. Answers are on a 4-point Likert scale (very much, moderately, somewhat, and not at all). The scale captures worry about fetal growth, health, and delivery method. The PRAS specifies three categories of anxiety levels (low, moderate, and high) with good reliability (Cronbach alpha = 0.78) [32]. We classified mothers as having high pregnancy-related anxiety if they chose “very much” to three or more questions on the PRAS and all other women served as the reference group. To assess depression at the mid-pregnancy visit, we administered the Edinburgh Postnatal Depression Scale (EPDS) [33], a 10-item questionnaire screening for depressive symptoms. Answers are on a 4-point Likert scale from 0 to 3. The EPDS is a validated screener for probable depression but it is not intended to diagnose clinical depression. The scale has been validated in pregnant women and has a sensitivity of 86% and a specificity of 78% for the diagnosis of depression [33, 34]. A score > 13 on the 0–30 scale indicates probable prenatal depression [35, 36].

To assess DNA methylation, we used umbilical cord blood collected at delivery and whole blood samples from early and mid-childhood visits. Technicians extracted DNA using the Qiagen Puregene Kit (Valencia, CA) and stored aliquots at − 80 °C until analysis. DNA underwent sodium bisulfite conversion using the EZ DNA Methylation-Gold Kit (Zymo Research, Irvine, CA). Samples were shipped to Illumina Inc. and analyzed for DNA methylation at > 485,000 CpG sites simultaneously using the Infinium Human Methylation450 BeadChip (Illumina, San Diego, CA).

We used a two-stage algorithm in which we randomized 12 samples to each chip and then randomly assigned eight chips to each of the 15 plates used to ensure balance by sex across chips and plates. We excluded samples as potentially mislabeled if they were mismatched on sex, genotype or were deemed to be low in quality. Background correction and dye-bias equalization was performed via the normal-exponential out-of-band (*noob*) method [37], and a β-mixture quantile intra sample normalization procedure (*BMIQ*) was applied to the data to reduce the potential bias that can arise from probe design [38]. For each CpG site, methylation is reported as average *β* value = *M*/(*M* + *U* + ε), where *M* and *U* represent the average fluorescence intensity from each probe corresponding to the methylated and unmethylated target CpG and ε = 100, a small quantity to protect against division by zero. Thus, the average *β* value is an interval-scaled quantity between zero and one interpreted as the fraction of DNA molecules whose target CpG is methylated across all nucleated cells. We excluded individual probes if they had non-significant detection *P* values (*P* > 0.05) for more than 1% of the samples. Additionally, non-CpG probes (probes for SNPs (rs) and methylated sites other than cytosine (ch)), probes in X and Y chromosomes, SNP-associated probes at either the single base extension or within the target region were removed for SNPs that have a minor-allele frequency of > 5%. Any probe with a SNP < 10 base pairs was excluded using annotation from the Bioconductor package IlluminaHumanMethylation450kanno.ilmn12.hg19 that utilized information from dbSNP. Previously identified non-specific and cross-reactive probes within the array along with polymorphic CpG sites were also excluded from the analysis [39]. We excluded individual probes with values greater than three times the interquartile range (IQR) from the 75th percentile or values less than three times the IQR from the 25th percentile to eliminate potential DNA methylation outliers. We used ComBat [40] to correct for technical variability from plate and scanner. We visually inspected the effectiveness of adjustment for batch using principal components before and after batch adjustment. We calculated the genomic inflation factor (*λ*) for all three EWAS to evaluate systemic biases.

After quality control, there were 372,563 loci for analysis. We logit-transformed methylation values on the *β* values (bounded between 0 and 100%) to *M* values prior to analyses as previously described to be more appropriate for the differential analysis of DNA methylation [41] but report results as %-change in DNA methylation for interpretability.

### Replication cohort: the Generation R Study

We pursued external replication of the top differentially methylated sites in Project Viva in an independent birth cohort study, the Generation R Study, based in Rotterdam, the Netherlands. For the Generation R Study, all pregnant women living in Rotterdam with an expected delivery date between April 2002 and January 2006 were asked to participate. In total, 9778 mothers were enrolled [42]. Cord blood DNA methylation was measured using Illumina Infinium HumanMethylation450 BeadChip (Illumina Inc., San Diego, USA).

Preparation and normalization of the HumanMethylation450 BeadChip array data were performed according to the CPACOR workflow [43] using the software package *R* [44]. In detail, the idat files were read using the *minfi* package. Probes that had a detection *P* value above background (based on the sum of methylated and unmethylated intensity values) ≥ 1 × 10^−16^ were set to missing per array. Next, the intensity values were stratified by autosomal and non-autosomal probes and quantile normalized for each of the six probe type categories separately: type II red/green, type I methylated red/green, and type I unmethylated red/green. Beta values were calculated as the proportion of methylated intensity value on the sum of methylated + unmethylated + 100 intensities. Arrays with observed technical problems such as failed bisulfite conversion, hybridization or extension, and arrays with a mismatch between sex of the proband and sex determined by the chr X and Y probe intensities were removed from subsequent analyses. Additionally, only arrays with a call rate > 95% per sample were processed further.

A subset of *N* = 999 mother-child pairs had complete information on maternal antidepressant use in pregnancy, and *N* = 969 had complete information on maternal depression and anxiety. Maternal prenatal depression and anxiety were assessed at 20 weeks of pregnancy with the Brief Symptom Inventory [45, 46]. This questionnaire comprises 53 items which provided nine scales of various psychiatric symptoms. The scale has a global index and includes two subscales for anxiety and depressive symptoms. The subscales for anxiety and depression contained six items each on a 5-point scale, from 0 to 4 where a higher score indicates a higher level of symptoms. Antidepressant use was reported during each trimester of pregnancy using a self-reported questionnaire. Use of SSRI was confirmed with prescription records from pharmacies with participant consent. These measurements have been previously described in detail [42, 47, 48].

### Statistical analyses

For each covariate in both discovery and replication cohorts, we calculated means and standard deviations (SD), or sample sizes and percentages, to describe the discovery and replication cohorts. In the discovery cohort, we performed epigenome-wide DNA methylation analyses on a CpG-by-CpG basis to assess DNA methylation differences at each site in cord blood relative to prenatal maternal exposure to (1) antidepressant prescription, (2) anxiety, and (3) depression compared to non-exposed infants. We used separate robust linear regression models with heteroskedasticity-consistent estimators to model the methylation levels of each individual CpG on the *M* value scale as the dependent variable and antidepressants, high pregnancy-related anxiety, and depression as predictors. We adjusted all regression models for variables selected a priori: maternal age, parity, self-reported race, smoking during pregnancy, body mass index (BMI), mode of delivery, education and infant sex, gestational age at birth, and nucleated cell-type proportions in cord blood (CD8+ T cells, CD4+ T cells, monocytes, natural killer cells, B cells, granulocytes, and nucleated red blood cells for cord blood analyses) estimated from the DNAm data using *minfi* [49]. Statistical significance for the CpG-by-CpG analyses was adjusted by controlling the false discovery rate at 5% (FDR < 0.05) for each of the three-independent epigenome-wide analyses. As secondary analyses, we tested for differentially methylated regions in relationship to antidepressant prescription, anxiety, and depression using DMRcate [50] with an FDR < 0.05.

Similarly, in the replication cohort, we fit a robust linear regression with each of the top CpGs from discovery as the outcome for each prenatal maternal exposure and adjusted for similar covariates as we had in discovery*.* We tested CpG sites associated with prenatal maternal antidepressant use that passed Bonferroni correction in the discovery cohort due to an early departure from the expected uniform distribution for this EWAS (Additional file 2: Figures S1-S2) and for depression and anxiety among significant differentially methylated sites that passed FDR < 0.05. In replication analyses, we deemed a *P* < 0.05 as statistically significant in addition to having the association be consistent in direction with the discovery cohort.

We also evaluated the persistence of associations in early and mid-childhood in Project Viva by carrying forward individual loci found to be associated with DNA methylation in cord blood analyses that also replicated in the Generation R Study. Persistence of DNA methylation differences was evaluated in peripheral blood samples collected during early and mid-childhood using multivariate robust linear regression models adjusting for the same covariates as cord blood models with the addition of child age at the time of the blood draw. We considered *P* < 0.05 as statistically significant for the persistence of epigenetic alterations in early or mid-childhood peripheral blood analyses. We also investigated unadjusted DNA methylation differences between exposed and unexposed children using boxplots and a Wilcoxon-rank sum test. We present the unadjusted distribution of DNA methylation levels in boxplots by antidepressant prescription given the relative small number of exposed infants. All analyses were carried out using the R statistical package, version 3.4.1 (www.r-project.org/).

### Blood-brain DNA methylation samples

We evaluated co-variation between blood DNA methylation and methylation levels of brain regions using publicly available data from the Gene Expression Omnibus (GEO) repository (GSE59685). Briefly, to generate reference data, investigators collected whole blood samples prior to death and matched those samples to postmortem samples from the prefrontal cortex, entorhinal cortex, superior temporal gyrus and cerebellum of *N* = 75 men and women (40–105 years old) [51]. They measured DNA methylation using the Illumina HumanMethylation450 BeadChip Array. Scatter plots and person correlation coefficients for the relationship between blood and brain DNA methylation was examined among sites that replicated in the external cohort.

## Additional files

Additional file 1: **Table S1.** Differentially methylated CpG sites in umbilical cord blood DNA associated with high anxiety and depression (FDR < 0.05 for the discovery cohort, Project Viva) and replication results from the Generation R Study. **Table S2.** Type of prenatal maternal antidepressants prescribed to the 14 unique participants in Project Viva. (DOCX 19 kb)

Additional file 2: **Figure S1.** Quantile-Quantile plots of observed vs expected *P* values and genomic inflation factor (λ) for Epigenome-Wide Associations of prenatal maternal A) prenatal antidepressants B) high pregnancy-related anxiety and C) depression. **Figure S2.** Manhattan plots for Epigenome-Wide Associations of prenatal maternal A) antidepressants B) high pregnancy-related anxiety and C) depression. (DOCX 852 kb)

## Abbreviations

BMI: Body mass index
CI: Confidence interval
EPDS: Edinburgh Postnatal Depression Scale
EWAS: Epigenome-wide association study
PRAS: Pregnancy-Related Anxiety Scale
SD: Standard deviation
SSRIs: Serotonin reuptake inhibitors
*ZNF575*: Zinc Finger Protein 575 gene
