# Supplementary material for: Prenatal maternal antidepressants, anxiety, and depression and offspring DNA methylation: epigenome-wide associations at birth and persistence into early childhood
Source: Clin Epigenetics. 2019 Mar 29;11:56. doi: 10.1186/s13148-019-0653-x (PMC6441191; doi:10.1186/s13148-019-0653-x)
Supplement: Supplementary file 1 — Table S1. Differentially methylated CpG sites in umbilical cord blood DNA associated with high anxiety and depression (FDR < 0.05 for the discovery cohort, Project Viva) and replication results from the Generation R Study. Table S2. Type of prenatal maternal antidepressants prescribed to the 14 unique participants in Project Viva. (DOCX 19 kb) [file 13148_2019_653_MOESM1_ESM.docx]

**Table S1.** Differentially methylated CpG sites in umbilical cord blood DNA associated with high anxiety and depression (FDR<0.05 for the discovery cohort, *Project Viva*) and replication results from the *Generation R Study*.

|  |  |  |  | **Discovery cohort**  ***Project Viva*** | | **Replication cohort**  ***Generation R Study* (n=969^c^)** | | | |
| --- | --- | --- | --- | --- | --- | --- | --- | --- | --- |
| **CpG** | **Chromosome** | **Genomic**  **Position** | **Gene** | **Mean (SD)**  **%-DNA methylation** | **Adjusted ^a^ % change in DNA methylation**  **(95 % CI)** | **Mean (SD)**  **%-DNA methylation** | **Adjusted ^b^ % change**  **in DNA methylation**  **(95 % CI)** | ***P*** |  |
| **Anxiety Models** (N=445) | |  |  |  |  |  |  |  |  |
| cg01305547 | 4 | 1893307 | *WHSC1* | 95.7 (1.5) | -1.11 (-1.63, -0.60) | 85.8 (3.2) | 0.47 (-0.10, 1.04) | 0.11 |  |
| cg01043865 | 5 | 37837978 | *GDNF* | 2.7 (0.5) | 0.37 (0.20, 0.55) | 11.4 (2.5) | 0.23 (-0.22, 0.68) | 0.31 |  |
| cg25278941 | 6 | 139795527 |  | 42.7 (8.7) | -2.19 (-3.26, -1.11) | 49.6 (8.4) | 0.56 (-0.29, 1.41) | 0.20 |  |
| cg14456161 | 10 | 3373441 |  | 98.7 (0.5) | -0.28 (-0.41, -0.14) | 90.7 (2.8) | -0.01 (-0.50, 0.48) | 0.97 |  |
| cg09303150 | 11 | 92928169 | *SLC36A4* | 87.6 (4) | -2.49 (-3.45, -1.53) | 77.5 (4.8) | 0.71 (-0.17, 1.59) | 0.12 |  |
| cg06826289 | 12 | 129468180 | *GLT1D1* | 91.9 (2.8) | 1.45 (0.84, 2.07) | 84.7 (2.7) | 0.17 (-0.33, 0.67) | 0.51 |  |
| cg14282941 | 14 | 50100316 | *C14orf104* | 1.4 (0.2) | 0.13 (0.07, 0.20) | 7.0 (1.6) | 0.07 (-0.29, 0.43) | 0.71 |  |
| cg19133221 | 17 | 47210279 | *B4GALNT2* | 2.2 (0.3) | 0.29 (0.17, 0.41) | 5.0 (1.7) | 0.16 (-0.12, 0.45) | 0.27 |  |
| cg03837680 | 17 | 77967529 | *TBC1D16* | 9.8 (4.1) | -1.16 (-1.67, -0.67) | 24.8 (6.1) | 0.18 (-0.72, 1.09) | 0.69 |  |
| cg23876292 | 18 | 57024517 | *LMAN1* | 94.3 (2.5) | -1.81 (-2.52, -1.12) | 84.8 (3.2) | -0.17 (-0.90, 0.55) | 0.64 |  |
| cg06616765 | 19 | 8570636 |  | 2.3 (0.5) | 0.33 (0.21, 0.45) | 10.1 (1.8) | -0.14 (-0.63, 0.35) | 0.57 |  |
| cg26327024 | 19 | 50412864 | *NUP62; IL4I1* | 98.9 (0.4) | -0.27 (-0.38, -0.16) | *NA* | *NA* | *NA* |  |
| cg25259296 | 21 | 43235862 | *PRDM15* | 97.0 (1.2) | -1.02 (-1.42, -0.62) | 86.1 (2.3) | -0.27 (-0.77, 0.23) | 0.29 |  |
|  | |  |  |  |  |  |  |  |  |
| **Depression Models** (N=402) | |  |  |  |  |  |  |  |  |
| cg06808585 | 1 | 160051087 | *KCNJ9* | 27.2 (4.6) | 3.10 (1.93, 4.17) | 37.3 (5.2) | 0.04 (-1.43, 1.52) | 0.96 |  |
| cg05245515 | 14 | 21467273 | *SLC39A2* | 95.8 (1.8) | -1.59 (-2.21, -0.97) | 88.5 (1.7) | 0.28 (-0.23, 0.79) | 0.29 |  |
| cg15264806 | 18 | 59000800 |  | 1.5 (0.2) | 0.14 (0.08, 0.19) | 1.9 (0.7) | 0.05 (-0.15, 0.25) | 0.63 |  |

Abbreviations: SD, standard deviation; CI, confidence interval, NA, Excluded from the replication cohort (*Generation R Study*) after standard quality control.

**^a^** Adjusted for maternal age at enrollment, parity, race/ethnicity, smoking (never, former and during pregnancy), BMI at enrollment, mode of delivery, education and infant sex, gestational age, and estimated cord blood nucleated cells (CD8, CD4, Mono, NK, B-cells, granulocytes and nRBCs).

^b^ Adjusted for the same covariates as above and included sample plate as an additional covariate.

^c^ Maximum sample size. Other CpGs had fewer samples that passed quality control standards and model n’s ranged from 957-969.

**Table S2.** Type of prenatal maternal antidepressants prescribed to the 14 unique participants in Project Viva.

| **Generic Drug Name** | ^a^ **Type of Antidepressant Prescribed** |
| --- | --- |
| Amitriptyline HCL | 1 |
| Bupropion HCL | 1 |
| Citalopram Hydrobromide | 2 |
| Desipramine HCL | 2 |
| Fluoxetine HCL | 3 |
| Paroxetine HCL | 2 |
| Sertraline HCL | 5 |

^a^ Some participants were prescribed different antidepressants during pregnancy. One study participant was prescribed both paroxetine and amitriptyline and another study participant was

prescribed both desipramine and sertraline
