# Supplementary material for: Prenatal maternal antidepressants, anxiety, and depression and offspring DNA methylation: epigenome-wide associations at birth and persistence into early childhood
Source: Clin Epigenetics. 2019 Mar 29;11:56. doi: 10.1186/s13148-019-0653-x (PMC6441191; doi:10.1186/s13148-019-0653-x)
Supplement: Supplementary file 2 — Figure S1. Quantile-Quantile plots of observed vs expected P values and genomic inflation factor (λ) for Epigenome-Wide Associations of prenatal maternal A) prenatal antidepressants B) high pregnancy-related anxiety and C) depression. Figure S2. Manhattan plots for Epigenome-Wide Associations of prenatal maternal A) antidepressants B) high pregnancy-related anxiety and C) depression. (DOCX 852 kb) [file 13148_2019_653_MOESM2_ESM.docx]

**Figure S1.** Quantile-Quantile plots of observed vs expected *p*-values and genomic inflation factor (λ) for Epigenome-Wide Associations of prenatal maternal **A**) prenatal antidepressants **B**) high pregnancy-related anxiety and **C**) depression


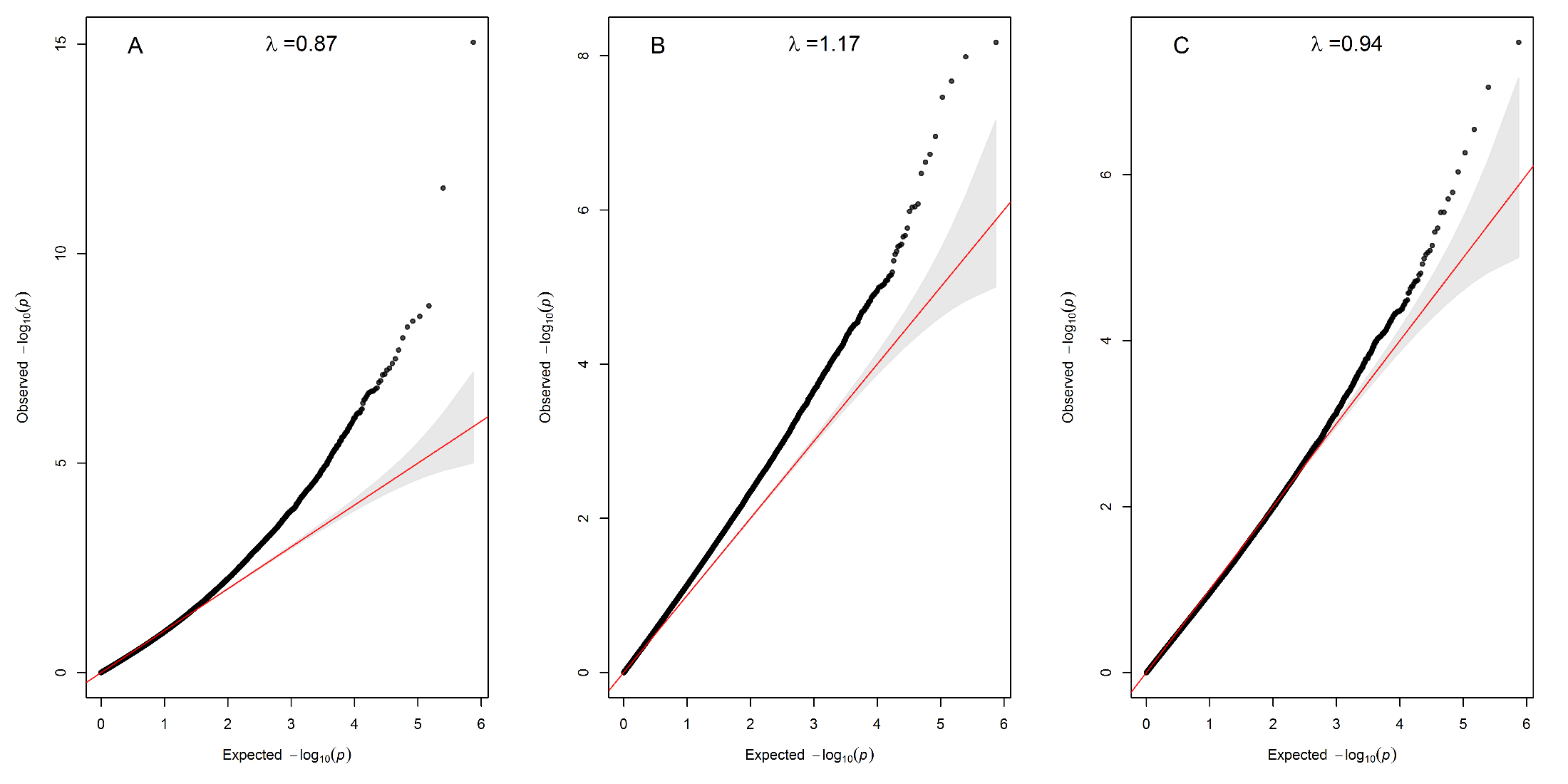


**Figure S2.** Manhattan plots for Epigenome-Wide Associations of prenatal maternal **A**) antidepressants **B**) high pregnancy-related anxiety and **C**) depression

**
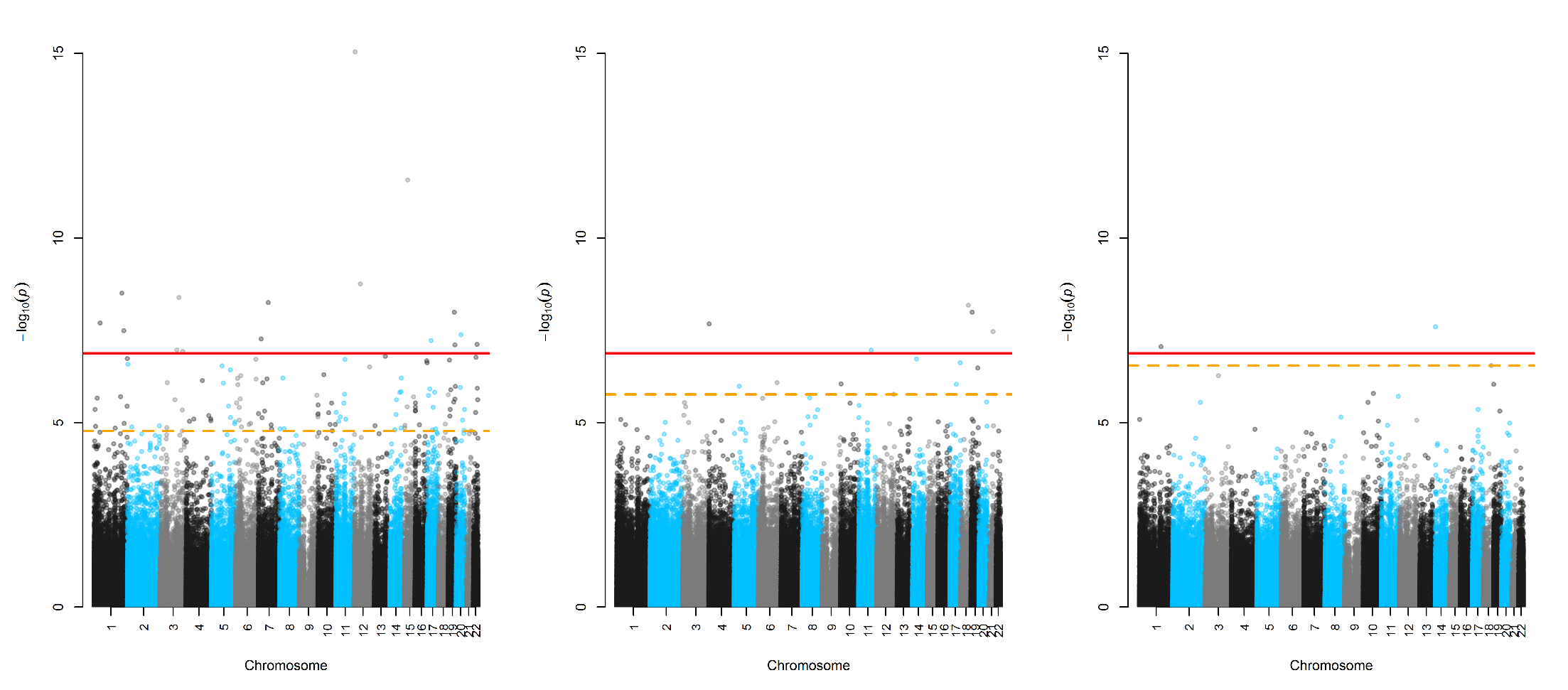
**
